# Toxicology and Analysis of Psychoactive Tryptamines

**DOI:** 10.3390/ijms21239279

**Published:** 2020-12-04

**Authors:** Sara Malaca, Alfredo Fabrizio Lo Faro, Alice Tamborra, Simona Pichini, Francesco Paolo Busardò, Marilyn A. Huestis

**Affiliations:** 1 Department of Excellence of Biomedical Sciences and Public Health, University “Politecnica delle Marche” of Ancona, Via Tronto 10, 60126 Ancona, Italy; smalaca@hotmail.com (S.M.); fabriziolofaro09@gmail.com (A.F.L.F.); tamborraalice@gmail.com (A.T.); 2 National Centre on Addiction and Doping, Istituto Superiore di Sanità, V.Le Regina Elena 299, 00161 Rome, Italy; simona.pichini@iss.it; 3 Institute of Emerging Health Professions, Thomas Jefferson University, 1020 Walnut St, Philadelphia, PA 19144, USA; marilyn.huestis@gmail.com

**Keywords:** tryptamines, new psychoactive substances (NPS), analytical methods, toxicology, psychedelics

## Abstract

Our understanding of tryptamines is poor due to the lack of data globally. Tryptamines currently are not part of typical toxicology testing regimens and their contribution to drug overdoses may be underestimated. Although their prevalence was low, it is increasing. There are few published data on the many new compounds, their mechanisms of action, onset and duration of action, toxicity, signs and symptoms of intoxication and analytical methods to identify tryptamines and their metabolites. We review the published literature and worldwide databases to describe the newest tryptamines, their toxicology, chemical structures and reported overdose cases. Tryptamines are 5-HT_2A_ receptor agonists that produce altered perceptions of reality. Currently, the most prevalent tryptamines are 5-methoxy-N,N-diisopropyltryptamine (5-MeO-DiPT), 5-methoxy-N,N- diallyltryptamine (5-MeO-DALT) and dimethyltryptamine (DMT). From 2015 to 2020, 22 new analytical methods were developed to identify/quantify tryptamines and metabolites in biological samples, primarily by liquid chromatography tandem mass spectrometry. The morbidity accompanying tryptamine intake is considerable and it is critical for clinicians and laboratorians to be informed of the latest data on this public health threat.

## 1. Introduction

Psychedelics are a diverse group of naturally occurring and synthetic drugs that induce distorted states of consciousness, perception, thinking and feeling [1]. Tryptamines share their core structure with the neurotransmitter serotonin, also named 5-hydroxytryptamine (5-HT). The effects of psychedelics, including those of tryptamines, are mediated by the 5-HT_2A_ receptor [2,3,4,5,6] but may also be modulated by interactions with other 5-HT receptors, monoamine transporters and trace amine-associated receptors [6,7,8,9,10,11,12,13,14]. Furthermore, Luethi et al. presented a brief report based on correlations between in vitro human 5-HT receptor affinities and their dose estimates [12], reporting that human doses for psychedelics were significantly correlated with 5-HT_2A_ and 5-HT_2C_ receptor binding but not with 5-HT_1A_ receptor binding. 5-HT_2A_ receptors expressed on neocortical pyramidal cells are involved in the psychedelic effects of tryptamines [3]. The activation of 5-HT_2A_ receptors increases cortical glutamate levels by a presynaptic receptor-mediated release from thalamic afferents. As serotonin receptor agonists, psychedelics can produce synesthesia and altered perceptions of reality, where senses usually experienced separately are combined. The status of control for different psychedelics varies, many are controlled under the Convention on Psychotropic Substances of 1971 (e.g., diethyltryptamine (DET), dimethyltryptamine (DMT), α-ethyltryptamine (α-ET)), although some synthetic psychedelics are not currently under international control.

Many countries report the use of psilocybin or magic mushrooms to the United Nations Office on Drugs and Crime (UNODC) [1]. The ranking of drugs by Member States, based on prevalence data from 123 countries, including 78 countries providing psychedelic data, suggests that the use of this drug class is currently ranked on average 5.3 from 2013–2017, being less of a threat than the use of cannabis, sedatives and tranquillizers, opioids and cocaine. From the available data on trends in the use of different substances from 2001–2017, the majority of countries reported no significant change in psychedelic use over time. Nonetheless, there are signs of an increase in the overall use of psychedelics in recent years, particularly from 2012 to 2016, with the number of countries reporting increases in the use of psychedelics greater than the number of countries reporting decreases. Furthermore, according to the 2019 Global Drug Survey (GDS) [15], psychedelics were consumed by 40% of drug users, with tryptamines the psychedelic class with the greatest increase in use. The psychedelics with the greatest prevalence of use were lysergic acid diethylamide (LSD) (17.5%), psilocybin or magic mushrooms (14.8%), DMT (4.2%), magic truffles (3.3%) and ayahuasca (1.1%) [16].

Several tryptamines occur naturally in a variety of plants, fungi and animals [1]. Some tryptamines are also chemically synthesized, with several DMT analogs, such as alpha-methyltryptamine (α-MT) and 5-methoxy-diisopropyltryptamine (5-MeO-DiPT), currently popular.

Several reviews report the toxicology of different NPS [17,18,19,20,21]; however, only two focused on tryptamines [22,23]. In 2015, Tittarelli et al. [22] presented a summary of all the currently available information on tryptamines and their derivatives, including pharmacology, chemical structures and effects related to routes of administration and toxicities. Data were provided for twelve tryptamines and/or derivatives and some intoxication reports. In the same year, Araújo et al. [23] provided an overview for the same classic tryptamines plus other derivatives, providing additional detail on the drugs’ toxicodynamics, preclinical physiological studies and adverse effects in humans. NPS and tryptamines are constantly evolving, with new drugs appearing rapidly onto the market. The morbidity associated with tryptamine intake is considerable and it is important for toxicologists to be informed of the latest data on this public health threat. Our aim is to present the latest tryptamine intoxication cases and new analytical methods to identify and quantify tryptamines in conventional and non-conventional biological matrices over the last five years.

## 2. Results

**Figure 1:**
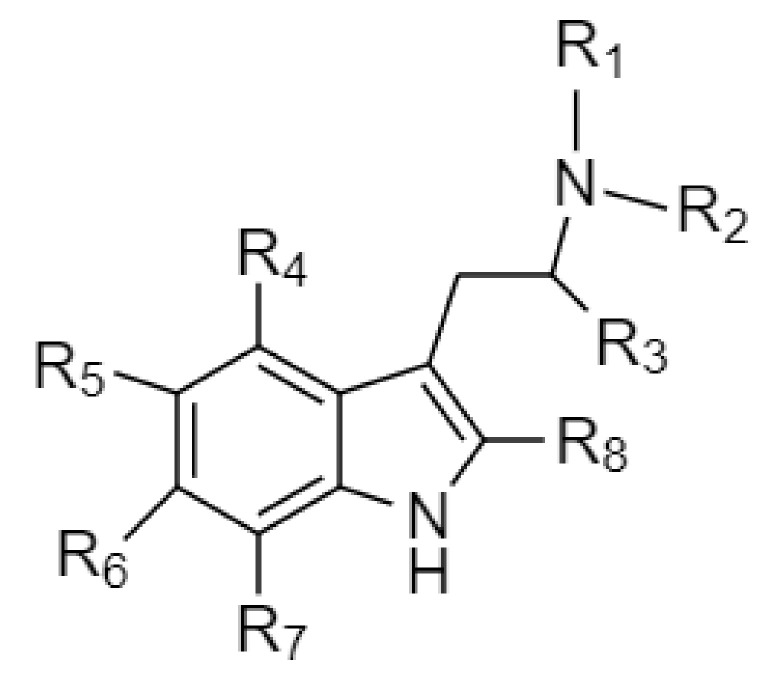
Tryptamines base structure.

Tryptamines are psychedelic drugs derived from decarboxylation of the amino acid tryptophan, which produces the typical indole ring [24], giving these compounds the name “indolealkylamines.” A compound’s chemical structure determines which receptors it can bind and activate, its absorption, distribution, metabolism and elimination and its effects. In fact, minor additions and modifications to the indolealkylamine backbone provide an endless supply of novel tryptamine structures, each with a unique pharmacology. Table 1 presents the most common and newest tryptamines and metabolites, with their common backbone structure and numerous ring substitutions. Tryptamines base structure is represented on Figure 1.

Some tryptamine structures facilitate crossing the blood brain barrier, with a rapid onset of highly potent effects and other structures prevent rapid metabolic degradation, increasing the duration of effects [25]. Some tryptamines and derivatives are potent and short-acting psychedelics, whose total duration of action is less than thirty min [25]. Such compounds must be taken parenterally or enterally to experience the psychedelic effects. MAO is a mitochondrial flavin-dependent enzyme that oxidatively deaminates serotonin and other biogenic and neuroactive amines and is present in the liver, gut and brain of humans and other mammals. If tryptamines are orally ingested, protection from peripheral degradation by a monoamine oxidase inhibitor (MAOI) may be necessary for activity. Consequently, MAOI generally increases the pharmacological effects of such bioactive amines.

### 2.1. Tryptamines of Natural Origin

• Dimethyltryptamine (DMT)

DMT shares psychedelic and hallucinogenic activity with lysergic acid diethylamide (LSD) and mescaline in terms of intensity and characteristics [22]. Common routes of DMT administration are oral, insufflation, intravenous (IV) and smoking [24]. The time course of DMT administered via inhalation of vaporized freebase or IV injection of a water-soluble salt is brief. The onset is rapid, with full effects noted within 2 min of administration and subjective effects fully resolving within 20–30 min [25]. Szára et al. also reported a rapid onset (2–5 min) of effects and a duration of action of 30–60 min following intramuscular (IM) administration of 0.2–1 mg/kg DMT [26]. These authors reported that 0.7 mg/kg IM DMT resulted in diarrhea, nausea and vomiting. Additionally, elevated heart rate, blood pressure and rectal temperature were reported by others following DMT administration [27]. Psychologically, DMT can cause short-term emotional distress and in some cases precipitate long-lasting psychosis. DMT is an agonist at the 5-HT_1A_, 5-HT_2A_ and 5-HT_2C_ serotonin receptors and at the sigma-1 receptor. DMT’s broad agonist activity includes modulation of physiological processes and regulation of inflammation through the sigma-1 and 5-HT receptors and changing immune responses through the sigma-1 receptor. IM effects are usually less intense than those following IV or inhalation. Oxidative deamination of DMT by monoamine oxidase (MAO) produces indole-3-acetic acid (3-IAA) and 3-indole-aceturic acid [28]. Other metabolic pathways include N-oxidation, N-demethylation and cyclization. DMT-N-oxide (DMT-NO), N-methyltryptamine (NMT), 2-methyl-1,2,3,4-tetrahydro-beta-carboline (2-MTHβC) were also identified as minor DMT metabolites [28].

DMT was first isolated from Mimosa hostiles, Mimosa tenuiflora and Mimosa root bark and is also present in Psychotria viridis leaves and Virola plants, all parts of the beverage Ayahuasca [25]. Ayahuasca is produced by mixing different plants by native populations of the basin of the Amazon river, suggested to be a drink with magic and curative powers. For the decoction preparation, the natives boil the bark or crushed stems of Banisteriopsis caapi together with other plants, including the leaves of Psychotria viridis, a member of the Rubiaceae family. The two plants are distinguished by the content of active compounds. Psychotria viridis contains DMT and Banisteriopsis caapi contains harmala alkaloids (Peganum harmala or Syrian Rue) harmine, tetrahydroharmine and harmaline [29]. DMT is a hallucinogen and the harmala alkaloids are MAO inhibitors that enhance DMT’s effects. Ayahuasca is used to treat depression, anxiety and alcohol, tobacco [30] and drug addiction [31]. DMT is also found as a minor alkaloid in the bark, pods and beans of Anadenanthera peregrina and Anadenanthera colubrina [32].

In 2015, Paterson et al. [33] reported an acute intoxication in Los Angeles, California of an unknown amount of smoked DMT by a 42-year-old male with no psychiatric history other than multiple substance use disorders. He was on an involuntary legal hold for bizarre and disorganized behavior that ultimately led to his hospitalization. During the interview in the emergency department (ED), he reported smoking cannabis and three weeks before hospitalization, he began smoking DMT. The subject was agitated, underweight and exhibited a marked disorientation to time. The patient received supportive therapy, including sedation with benzodiazepines. After 21 days hospitalization, he was discharged without complications. The urine toxicology analysis was performed 5 days after ED admission and resulted positive only for benzodiazepines and negative for DMT.

In 2017, Bilhimer et al. [34] reported an acute intoxication involving DMT in the U.S. A 25-year-old male with a history of mental illness had strong hallucinations and suicidal thoughts after injecting DMT intravenously. The subject had dilated pupils, tachycardia (88 bpm) and systolic and diastolic blood pressure of 116/71 mm Hg. His urine was immunoassay positive for amphetamines and DMT at a concentration greater than 2000 ng/mL.

• Psilocybin and psilocin

Psilocybin (4-Phosphoryloxy-N,N-dimethyltryptamine) and psilocin (4-Hydroxy-N,N-dimethyltryptamine or 4-OH-DMT), are contained in about 190 species of Psilocybe mushrooms but the most well-known varieties are Psilocybe cubensis, Psilocybe semilanceata and Psilocybe Mexicana. Psilocybin is a 4-substituted indoleamine that is dephosphorylated to psilocin, its pharmacologically active metabolite [35]. Albert Hofmann [36] first isolated and identified psilocin from the Psilocybe Mexicana mushroom in 1958. Psilocybe cubensis contains the highest concentrations of these two tryptamines and is available frozen or as a dry powder or capsule [36]. In addition to its natural origin, synthetic psilocin is available fresh or treated/preserved (dried or cooked). Psilocin is highly unstable in solution and in the presence of oxygen and alkaline pH, it forms bluish and black degradation products. This tryptamine is an isomer of bufotenine, differing only in the position of the hydroxyl group [35]. Psilocin is orally active with a duration of action of 4–6 h [25]. Psilocin undergoes oxidative deamination and forms the minor metabolite 4-hydroxyindole acetic acid (4-HIAA). This tryptamine is also subject to phase II metabolism to the O-glucuronide, the main metabolite detected in human urine. In recent years, fungis’ sclerotia, commonly called “magic truffles,” frequently supply the psychoactive Psilocybe alkaloids, as Psilocybe sclerotia are not specifically included in the laws banning the sale, the purchase and the use of such substances [37]. Terms for magic truffles include The Philosopher Stones, Space truffles, Sclerotia Stones or Sclerotia. Psilocybin mushroom ingestion produces hallucinations as early as 10 min post ingestion of 1–2 mg dried mushrooms and typically lasts from 4–12 h. Common symptoms include dizziness, giddiness, nausea, weakness, muscle aches, shivering, anxiety, restlessness and abdominal pain [38,39]. In 2018, Honyiglo et al. described the death of an 18 year old male in France who jumped from the second floor and died following “hallucinogenic mushroom” intake [40]. The intoxication was confirmed by identification and quantification of psilocin in cardiac and femoral blood (67 and 60 ng/mL respectively), urine (2230 ng/mL), bile (3102 ng/mL) and vitreous humor (57 ng/mL). Gas chromatography time-of-flight detection with electron impact ionization (GC-EI-TOF) was the analytical technique employed for identification and quantification.

Currently, there is great interest in psilocybin in combination with psychotherapy to treat psychiatric disorders like anxiety, depression and addiction to nicotine and drugs [41,42]. For example, Grob et al. dispensed 0.2 mg/kg oral psilocybin, with a niacin placebo control to advanced-stage cancer patients with anxiety [43]. There was a significant reduction in anxiety at 1 and 3 months after treatment based on patients’ Speilberg State-Trait Anxiety Inventory (STAI). Carhart et al. [41] administered psilocybin to treat 12 patients with moderate-to-severe, unipolar, treatment-resistant major depression with two oral doses of psilocybin (10 mg and 25 mg, 7 days apart) in a supportive setting. In addition, two randomized blinded controlled clinical trials demonstrated significant long-term reductions in anxious depressed mood after psilocybin treatment [42,44]. Essential distress also decreased and quality of life improved in terminally ill cancer patients after a single oral dose of psilocybin. An open-label pilot study provided psilocybin in combination with cognitive behavioral therapy to 15 treatment-resistant tobacco/nicotine-dependent smokers [45]. Smoking abstinence was observed in 67% of patients at follow-up, documenting that psilocybin was more effective than the most successful FDA-approved medication, varenicline [38]. Finally, a recent open-label pilot study investigated psilocybin for the treatment of 10 alcohol-dependent individuals, with alcohol use decreasing dramatically after the first psilocybin administration [46].

• 5-Methoxy-N,N-dimethyltryptamine (5-MeO-DMT)

5-MeO-DMT is a natural tryptamine [25] that requires the presence of a MAOI to produce psychedelic effects [22]. 5-MeO-DMT is ingested but unpublished reports describe inhalation as a common mean of consumption with effects appearing within 60 sec and lasting 5–20 min [46]. Effects include auditory, visual and time perception distortions, emotional experiences, memory impairment, asthma (12%), high blood pressure (9%) and chronic fatigue syndrome (8%) [47]. There is also evidence that some people use 5-MeO-DMT for treating psychiatric conditions, including depression, anxiety, post-traumatic stress disorder and problematic substance use [47,48]. Tryptamine derivates like DMT, 5-OH-DMT and 5-MeO-DMT are metabolized by MAO-A that catalyzes an oxidative deamination forming IAA [49,50,51].

Brush et al. reported a poisoning related to 5-MeO-DMT and harmaline ingestion [22,52]. The victim was a 17 year old male found collapsed after insufflation of 15–20 mg 5-MeO-DMT. GC/MS analysis of urine only confirmed the presence of both harmaline and harmine. At ED admission, the patient had hypertension and tachycardia (186 bpm) and was hyperthermic (40.7 °C). After administration of 2.5 mg lorazepam, the symptoms began to resolve and the patient was discharged without complication.

Sklerov et al. described the death of a 25 year old white male found unresponsive in a national park following ingestion of 5-MeO-DMT. Toxicological analyses revealed the presence of 5-MeO-DMT in blood, urine, gastric contents, bile, kidney, brain and liver. Moreover, the heart blood sample contained DMT (0.02 mg/L), 5-MeO-DMT (1.88 mg/L), tetrahydroharmine (0.38 mg/L), harmaline (0.07 mg/L) and harmine (0.17 mg/L). The medical examiner pronounced the death as due to psychedelic amine intoxication [22,53].

### 2.2. Tryptamines of Synthetic Origin

#### 2.2.1. Ring Unsubstituted Tryptamines

• Αlpha-methyltryptamine (α-MT)

α-MT is a psychedelic tryptamine sold as a white crystalline powder [54]. It was first developed in 1960 as an antidepressant in the Soviet Union but its marketing was unsuccessful. α-MT’s duration of effects is 8–14 h if ingested and 3–6 h when inhaled. α-MT effects are similar to those of 3,4-methylenedioxymethamphetamine (MDMA); both are empathogens and strong stimulants [54]. α-MT strongly inhibits re-uptake and release of monoamines dopamine, serotonin and norepinephrine in mouse brain synaptosomes, also being a strong MAO inhibitor.

In September 2004, the U.S. Controlled Substances Act placed α-MT in Schedule I. α-MT is a hallucinogen [54] and a stimulant with an alpha methyl group similar to amphetamine [55]. α-MT is generally available as a powder, tablet or capsule with oral doses of 5–10 mg producing stimulation, 20–30 mg hallucinogenic effects for up to 24 h, 60–80 mg considered a “strong” dose and up to 150 mg α-MT was previously self-administered. Generally, 5–20 mg free base is smoked. Snorting or insufflation is an infrequent route of administration due to burning of the mucosa and a bad odor. Inadequate solubility and lack of increased pharmacological effects limits IV administration [56]. The most frequent adverse effects described in the literature are anxiety, muscle tightness, vomiting, hyperthermia, mild increase in blood pressure or respiratory rate, tachycardia, salivation, nausea, impaired coordination, nervousness and restlessness [56,57].

Recently, Holstege et al. reported the intoxication and ED admission of a 21-year-old man following α-MT ingestion [22,58]. He was hypertensive (BP 183/93 mmHg, heart rate 52 bpm), had a high fever, felt disoriented, nervous and trembling. After medical treatment, the patient was discharged without complication. No analytical confirmation was performed. In the same year, another α-MT intoxication involved a 17-year-old male found naked and hallucinating [22,59]. In the ED, he was hyperthermic and tachycardic (160 beats/min), presented with fever (37.3 °C), sweating and reactive 6–7 mm mydriatic pupils. α-MT was identified in his urine by high-performance liquid chromatography coupled with mass spectrometry (HPLC-MS). He was discharged with supportive therapy including benzodiazepines. Boland et al., reported the first known α-MT death of a 22 year old male found unresponsive by his roommate, who said he consumed 1 g α-MT 12 h prior. Iliac vein blood, gastric contents, liver and brain were all α-MT positive at 2.0 mg/L, 9.6 mg/kg; 24.7 mg/kg and 7.8 mg/kg, respectively [22,60].

There are few α-MT pharmacokinetic data. Hydroxylation or oxidation of the indole ring at the 2-, 6- and/or 7-positions was observed in male Wistar rats [61]. 2-oxo-α-MT, 6-hydroxy-α-MT, 7-hydroxy-α-MT and 10-hydroxy-α-MT metabolites were identified in rat urine. Other rat α-MT metabolic pathways include deamination to indole-3-acetone followed by oxidation to indole-3-carboxylic acid [61]. Also, an isomer of α-MT, 5-(2-aminopropyl) indole (5-IT, 5API), appeared on the European drug market in 2011 [62].

• Alpha-ethyltryptamine (α-ET)

α-ET was first synthesized in 1947 as a potential synthetic precursor to the β-carbolines but appeared on the clandestine market as an antidepressant (Monase) in the mid-1980s [63]. In 1986, Daldrup et al. described its first illicit use in Germany [64] and in 1993 the U.S. Drug Enforcement Administration (DEA) included it on the Schedule I list [65]. α-ET may produce serotonin neurotoxicity [66] but the exact mechanism is not understood. Serotonin neurotoxicity was produced by the combined administration of a non-neurotoxic serotonin releasing agent and the dopamine releasing agent S-amphetamine [67]. Other authors added that α-ET was the first tryptamine derivative, indeed, the first non-phenylisopropylamine, shown to produce stimulus effects similar to those of MDMA on unconditioned motor behavior in rats [62,68]. Like α-MT, other simple synthetic tryptamines, such as α-ET, possess a methyl group on the alpha carbon, providing protection from MAO metabolism [56]. α-ET has a duration of action of 6–8 h when taken orally [23].

Morano et al. described a fatal intoxication involving α-ET ingestion [22,69]. The victim’s autopsy revealed bilateral “pulmonary edema and generalized visceral congestion with some epicardial petechiae.” α-ET intake was confirmed in blood (5.6 mg/L), urine (80.4 mg/L), vitreous (2.4 mg/L), bile (22.0 mg/L), stomach contents (52.9 mg/g), liver (18.3 mg/g), kidney (24.0 mg/g) and brain (16.2 mg/g). Daldrup et al. described another fatal case in which a psychotic man presented with agitation and hyperpyrexia [22,64]. Blood toxicological analysis identified α-ET at 1.1 mg/L, with the cause of death listed as malignant hyperthermia with α-ET contribution.

• Diethyltryptamine (DET)

DET is the ethyl analogue of DMT. It is orally active because the ethyl group prevents MAO degradation. The active dose is 50–100 mg, with psychoactive effects lasting 2–4 h [70,71]. DET’s most common effects include slight generalized tremors to gross athletic movements, visual distortion, hypersensitivity to light, visual hallucinations, auditory perceptual distortions and olfactory hallucinations [72].

• N-methyl-N-ethyltryptamine (MET)

MET is structurally related to DMT but little scientific information is available on its pharmacology or toxicity in humans. Inhalation of 15 mg freebase MET or 80–100 mg oral freebase MET produce effects [73]. Anecdotal reports describe the most common physical and psychological effects as physical euphoria, tactile enhancement, increased heart rate and blood pressure, muscle cramps, teeth grinding, pupil dilation, hallucinations and cognitive effects [73].

• N-methyltryptamine (NMT)

NMT is the product of methyl group additions to tryptamine by the enzyme indolethylamine-N-methyltransferase [74]. In 2014, Gardner et al. demonstrated that NMT was a teratogen in pregnant rats, producing fetal skeletal malformations and cleft palates [75]. MAO metabolism produces its IAA metabolite.

• Dipropyltryptamine (DPT)

DPT is a synthetic tryptamine with a crystalline hydrochloride salt and an oily or crystalline base, first reported in 1973 as a treatment for alcoholism [76]. DPT is a hallucinogen, also known as “The Light.” DPT increases the intensity of music and color, with pleasant flashes of light and sparkles, causing one to lose one’s ego and producing visual experiences [22]. DPT was an adjunct to psychotherapy in the 1960s and 1970s but few peer-reviewed experimental studies were conducted [76]. Anecdotal internet reports describe it as a hallucinogen in humans following oral administration of 100–250 mg. Adverse physical and psychological “positive” effects include auditory hallucinations, music enhancement, stimulation, euphoria and relaxation, as well as “negative” effects such as paranoia, psychosis, anxiety, nausea, dizziness, increased heart rate and general tremor [77].

DPT strongly inhibited 5-HT reuptake into rat synaptosomes [12] and had moderate affinity and partial agonism at the human 5-HT_1A_ receptor [78]. In 2008, Fantegrossi et al. reported that DPT elicited head-twitches in mice and rats suggesting that a primary site of action for DPT is the 5-HT_2A_ receptor [79]. Dailey et al. described a DPT intoxication case of a 19-year-old female [22,80]. The patient arrived at the ED with tachycardia (200 bpm) and psychomotor agitation and was immediately sedated with 3 mg lorazepam. No analytical confirmation was performed but a vial of DPT labelled “for research purposed only” was found at the scene.

• Diisopropyltryptamine (DiPT)

DiPT is a synthetic hallucinogen, closely related structurally to DMT, a 5HT_2A_ agonist and a partial 5HT_1A_ agonist that inhibits the serotonin transporter and vesicular monoamine transporter [81]. DiPT produces short-term visual and auditory hallucinations including hearing voices and bizarre dream states at high doses and auditory distortion with deep tones at low doses [82].

#### 2.2.2. 4-Substituted Tryptamines

• 4-Hydroxy-N-methyl-N-ethyltryptamine (4-OH-MET)

4-OH-MET is one of the most common synthetic tryptamines available online. 4-OH-MET, synthesized by Shulgin et al. [82], has effects lasting 4–6 h. 4-OH-MET can produce a wide range of effects including prickling, decreased ability to move and later anxiety, nervousness, paranoia, tiredness and insomnia [31].

• 4-Hydroxy-N,N-dipropyltryptamine (4-OH-DPT)

4-OH-DPT is the 4-hydroxylated DPT derivative first synthesized by Shulgin et al. [82]. 4-OH-DPT is a light beige or white powder [54] that acts as a 5-HT_2A_ partial agonist. 4-OH-DPT also shares structural similarity with psilocin [83]. Effects are dose dependent, with onset at 15–45 min and duration of 5–8 h. According to user reports, synthetic 4-OH-DPT produces visual effects and hallucinatory states [84].

• 4-Acetoxy-N,N-dipropyltryptamine (4-AcO-DPT)

4-AcO-DPT is the 4-acetoxy derivative of DPT, is structurally similar to 4-AcO-DMT and 4-acetoxy-N, N- diisopropyltryptamine (4-AcO-DiPT) and is available as a white powder [54]. It is available online and typically administrated orally or by insufflation. Its effects are dose dependent and include hallucination, dissociation, confusion and flashbacks, as reported on Erowid by a 4-AcO-DPT user [85].

• 4-Acetyloxy-N,N-diallyltryptamine (4-AcO-DALT)

4-AcO-DALT is a tryptamine derivative structurally linked to DALT and 5-MeO-DALT [54]. Users report taking 15–30 mg doses, with effects similar to those of hallucinogenic mushrooms or 4-AcO-DMT [85]. Effects lasted 1 h and were similar to those following mushroom intake, including mental and visual effects and a slight cough and gagging [85].

• 4-Acetoxy-N-methyl-N-ethyltryptamine (4-AcO-MET)

4-AcO-MET, also known as matacetin, is a homolog of O-acetylpsilocin (4-AcO-DMT), an acetate ester of 4-OH-MET and a homologue of 4-AcO-DMT. It is also a structural and functional psilocin analogue and the 4-hydroxyl MET analogue [70].

Other synthetic 4-substituted tryptamines for which no scientific data are available are: 4-Hydroxy-diethyl-tryptamine (4-OH-DET), 4-OH-DiPT, 4-Hydroxy-N-methyl-N-isopropyltryptamine (4-OH-MiPT), 4-Hydroxy-N-methyl-N-propyltryptamine or meprocin (4-OH-MPT), 4-Methoxy-N-methyl-N-isopropyltryptamine (4-MeO-MiPT), 4-Methoxy-N,N-dimethyltryptamine (4-MeO-DMT), 4-Hydroxy-N,N-tetramethylenetryptamine (4-OH-pyr-T) and their acetic acid derivatives (e.g., 4-Acetoxy-N,N-dimethyltryptamine (4-AcO-DMT), 4-Acetoxy-N,N-diethyltryptamine (4-AcO-DET) and 4-Acetoxy-N,N-diisopropyltryptamine (4-AcO-DiPT).

#### 2.2.3. 5-Substituted Tryptamines

• Bufotenine or 5-Hydroxy-N,N-dimethyltryptamine (5-OH-DMT)

5-OH-DMT is a serotonin derivative found in the skin secretions of some Brazilian Rhinella toads [86], as well as in plants of the Leguminosae family and psychoactive mushrooms (Amanita muscaria) [87]. When Bufo species are ingested, toxicity ensues, including hemiparesis, muscle jerking and twitching, convulsions, altered mental status, slurred speech, headache, nausea, vomiting, severe dyspnea and death [88,89,90]. Furthermore, effective dosage, route of administration and psychedelic effects are widely known and reported in the literature [91,92]. It is metabolized by the enzyme monoamine oxidase A (MAO-A) that catalyzes an oxidative deamination forming IAA [86].

• 5-Methoxy-α-methyltryptamine (5-MeO-α- MT)

At low dosages (15–30 mg orally, 5–20 mg smoked), 5-MeO-α-MT produces powerful hallucinations [60]. Depending upon the individual, negative effects, including death, are more typical at higher dosages (80–100 mg oral 5-MeO-α-MT) [6,59]. Neurologic manifestations that may occur include agitation, restlessness, confusion and lethargy. Physical manifestations include vomiting, papillary dilation, jaw clenching, tachycardia, mydriasis, salivation, diaphoresis and mild elevations in blood pressure, temperature and respiratory rate may also occur [59].

• 5-Methoxy-N, N-dipropyltryptamine (5-MeO-DPT)

5-MeO-DPT is a 5-MeO-DMT structural isomer available as a white powder [54]. This molecule is orally active, with dose-dependent effects. The most common reported effects included dizziness, increased heart rate, tremor, anxiety and agitation.

• 5-Methoxy-N, N-diisopropyltryptamine (5-MeO-DiPT)

“Foxy Methoxy” or 5-MeO-DiPT is a well-known psychedelic tryptamine in the illicit market [93]. The common routes of administration are oral, smoked and insufflations [94]. Noworyta-Sokołowska et al. showed that 5-MeO-DIPT at doses of 5, 10 and 20 mg/kg induced head-twitch, hallucinations, vomiting and tachycardia in rats [94]. Moreover, long-term exposure to 5-MeO-DIPT resulted in development of post-hallucinogenic perception disorder. As described by Shulgin et al., users feel the effects of 6–10 mg 5-MeO-DiPT 20 to 30 min after administration for 3–6 h [95]. 5-MeO-DIPT is metabolized by five pathways, including hydroxylation, N-dealkylation [96] and reactions of O-demethylation, with the N,N-diisopropyl groups converted to 5-hydroxy-N,N-diisopropyltryptamine (5-OH-DiPT) that is further partly sulfated and glucuronidated [22,97].

The literature contains several reports of 5-MeO-DiPT intoxications. A 23-year-old male arrived at the ED with paranoia and sensory distortion after ingesting 5-MeO-DiPT. 5-MeO-DiPT intake was confirmed in blood and urine (0.14 and 1.6 μg/mL, respectively), suggesting consumption shortly before intoxication [22,98]. Smolinske et al. investigated an intoxication case of a psychotic 19-year-old male following ingestion of “Foxy,” transported to the ED and immediately treated with lorazepam [22,99]. Urine analysis was positive for phencyclidine and cocaine but was negative for 5-MeO-DiPT. In 2003, Meatherall et al. described the intoxication of a 21-year-old man found in a state of confusion [22,100]. At ED admission, the patient was alert and oriented, with the only clinical observation his inability to move his limbs. After 3.5 h, he was discharged from the hospital. Gas chromatography-mass spectrometry (GC-MS) analysis of his urine revealed 1.7 µg/mL 5-MeO-DiPT, codeine, nicotine, cotinine, acetaminophen and caffeine.

• 5-Methoxy-N,N-diallyltryptamine (5-MeO-DALT)

5-MeO-DALT was first synthesized by Shulgin et al. in 1980 [95] and was seized by police in 2006 as a “plant fertilizer” or “plant food” [29]. Active oral doses ranged from 12–20 mg [101], with administration by the oral, nasal insufflation and IV routes. This drug produced dose-dependent effects 15 to 30 min after oral administration that lasted for 2 to 4 h. Adverse effects included vasoconstriction, increased blood pressure, rapid heartbeat, headache, sweating, dilated pupils, bruxism, anxiety and nausea. Meatherall et al. reported a fatal road crash following ingestion of 350 mg 5-MeO-DALT by a 26 year old male [22,100]. Toxicological findings included the presence of 5-MeO-DALT but there were no data on drug quantification.

Michely et al. [102] identified 5-MeO-DALT phase I and II metabolites by liquid chromatography–high-resolution mass spectrometry (LC–HRMS/MS). Metabolites were primarily derived from hydroxylations, oxidations to oxo metabolites, N-dealkylations and O-demethylations.

• 5-Methoxy-N-methyl-N-isopropyltryptamine (5-MeO-MiPT)

5-MeO-MiPT or “moxy” was marketed as a “plant fertilizer.” Oral doses ranged from 1–3 mg (light), 3–8 mg (common) and 8–12 mg (strong), with typical 10–20 mg doses if inhaled [22,103]. The principal effects lasted 3–7 h and included a general heightening of awareness, mild euphoria, psychedelic visual effects, such as enhanced colors but also anxiety, nausea, confusion and paranoia. Repke et al. studied if the effects of the drug would differ depending upon the route of administration [104]. If ingested the effects were stimulating, with visual hallucinations prevailing. 5-MeO-MiPT metabolism studied by LC-HRMS/MS identified six phase I metabolites following N-demethylation, O-demethylation, demethylation and hydroxylation and N-oxide formation and hydroxylation of the parent compound and N-O-bis-demethylation of the metabolite 5-OH-MiPT [105].

In 2019, Grafinger et al. reported that a 32 year old male went to the ED after the ingestion of an “LSD-like substance” [106]. The cause of the intoxication was attributed to the combined use of psychoactive substances. 5-MeO-MiPT was identified by LC-HR-MS/MS and quantified by liquid chromatography coupled with mass spectrometry in tandem (LC-MS/MS) at 160 ng/mL in blood and 3380 ng/mL in urine. The most abundant metabolites were identified as 5-methoxy-N-isopropyltryptamine (5-MeO-NiPT), 5-hydroxy-N-methyl-N-isopropyltryptamine (5-OH-MiPT), 5-methoxy-N-methyl-N-isopropyltrypt-amine-N-oxide (5-MeO-MiPT-N-oxide) and hydroxy-5-methoxy-N-methyl-N-isopropyl-tryptamine (OH-5-MeO-MiPT).

Shimizu et al. reported an acute intoxication in a 27-year old male from 5-MeO-MiPT and methylone use [107]. At the ED, he had psychomotor agitation, was sweating, had pyrexia (37.8 °C) and tachycardia (150 bpm) and was hypertensive. The unknown powder that the man took was analyzed by high-performance liquid chromatography (HPLC) and GC-MS to determine the intoxicating drug(s). The powder contained 60% methylone (120 mg), a synthetic cathinone and 38% 5-MeO-MiPT (76 mg). No analytical confirmation was performed and rapid drug screening tests did not show immunoreactivity for other common drugs of abuse in urine and gastric fluid samples. He was discharged the day after admission without complications.

• 5-Methoxy-N-methyl-N-ethyltryptamine (5-MeO-MET)

5-MeO-MET is a tryptamine structurally similar to 4-OH-MET and 4-AcO-MET [54]. This drug is available in powder and crystal forms, with the common routes of administration ingestion and insufflation. A drug-forum site reported a “threshold” dose of 1–4 mg, a “normal” dose 5–8 mg and a “strong” dose 9–10 mg [108]. The onset of effects occurred at 1 min, with a limited duration of action of only 40 min. The main adverse effects reported by users include euphoria, hallucinations, visual alterations and anxiety.

• 5-Methoxy-N, N-diethyltryptamine (5-MeO-DET)

5-MeO-DET is an indolealkylamine structurally similar to DMT [105], with little data on its behavioral effects. In 2011, Gatch et al. observed stimulant effects and increased locomotor activity in rats [81]. After intraperitoneal and intraocular injection of 0.05 to 10 mg/kg 5-MEO-DET, effects occurred within 30 min and lasted 80 to 90 min.

• 5-Methoxy-N,N-trimethyltryptamine (5-MeO-TMT)

5-MeO-TMT is a psychedelic tryptamine first synthesized by Shulgin et al. [82] that is smoked or orally administered [104]. Toxicological information is still scarce but users suggest the oral dose is 75–150 mg but no duration of effects was reported. The effects include euphoria, visual distortions and difficulty in sleeping.

### 2.3. Analytical Methods to Determine Tryptamines and/or Metabolites in Conventional and Non-Conventional Biological Matrices

From 2010 to 2020, twenty-nine analytical methods were presented to determine tryptamines and metabolites. Table 2 summarizes the analytical methods reported for the determination of tryptamines in biological matrices, including information on sample size, sample preparation, instrumentation and validation parameters.

Different biological matrices were employed for forensic purposes including conventional matrices urine [102,112,114,118,121,122,123,125,126], blood [110,113,114,115,117,118,119,121,123], plasma [109,111,112,118] serum [112] and unconventional matrices oral fluid [120] and hair [116,124]. Protein precipitation is a simple sample preparation method, providing sufficient extraction efficiency while simplifying extraction and preparation time. Different protein precipitation methods [115,117,119,121] include different solvents for blood extraction but methanol is the most common solvent. Different sample preparation techniques include protein precipitation [113,116,117,119] for urine, serum and blood samples, acid hydrolysis [102,121,122] for urine, centrifugation [102,111,112,114,115,116,117,119,121,122,123,124,126] for blood, plasma and urine samples, washing [109,114,116,125] for plasma, blood and hair samples, enzymatic hydrolysis for urine samples [112], solid phase extraction (SPE) [109,110,111,114], liquid-liquid extraction (LLE) [114,120], dispersive liquid-liquid microextraction (DLLME) [115] and a solvent extraction for dried blood spots (DBS) [113]. Tryptamine is a relatively labile analyte but it remains stable in an acidic environment. Some analytical methods described the use of acidic solvents to extract drugs from blood.

Most analytical methods included chromatographic separations and mass spectrometry detection. LC-MS/MS [109,111,112,113,114,115,116,117,119,123,124], LC-HRMS/MS [102,122], GC-MS [102,118,122,123], LC-MS [118,123] and ultra-performance liquid chromatography-high resolution mass spectrometry (UHPLC-HRMS/MS) [121] analytical methods are most common for forensic toxicology purposes. Different approaches utilized triple quadrupole full-scan mode [102,126], triple quadrupole multiple reaction monitoring (MRM) [109,110,111,113,116,119,124] with data dependent acquisition (DDA) [102] and photodiode array acquisition PDA [118], single quadrupole time of flight (TOF) [121,125]. Even with powerful analytical instrumentation, these substances’ identification can be missed for many reasons including the fact that the reference mass spectrum may not be available in libraries for full-scan mode and fragments generated in single ion monitoring (SIM) mode show nondescript fragmentation patterns [22].

Low limits of detection (LOD) are key for identifying tryptamines; two analytical methods achieved LODs of 0.10–0.15 ng/mL for 5-MeO-DiPT, with the same sample volume and LC-MS/MS technology but different sample preparation techniques [117,119]. Michely et al. had LODs of 10–50 ng/mL for DALT and 5-MeO-DALT by GC-MS, with a 2.5 mL urine sample [102] but later achieved LODs of 1–5 ng/mL using LC-HRMS/MS and fifty times lower sample volume [122]. Also, Martin et al. determined psilocin in 500 µL plasma with a 0.1 ng/mL LOD [111], whereas in their more recent method they present an LOD of 0.05 ng/mL using 1 mL of the same matrix with a different sample preparation [112]. Fagiola et al. detected DMT in blood with a 2.5 ng/mL LOD [123], while Adamowicz et al. identified DMT with a 0.88 ng/mL LOD in a smaller blood volume but a longer sample preparation procedure [117], both with LC-MS/MS. For 5-MeO-DMT, Fagiola et al. [123] had a similar LOD as Adamowicz et al. (2.50 and 2.61 ng/mL, respectively) but the first author employed a higher sample volume [117]. More rigorous sample preparation can reduce background noise and improve detectability. Adamowicz et al. accomplished a lower 4-MeO-DiPT LOD compared to that of Vaiano et al. [119] with the same sample volume and mass spectrometer.

Yan et al. [126] analyzed urine for 5-MeO-DiPT and metabolites using a GC-Orbitrap-MS method characterized by LODs of 1 ng/mL and a concentration range of 1–2.8 ng/mL in five authentic urine samples. In this study the authors also identified two metabolites, 5-OH-DiPT and 5-MeO-DiPT but without presenting their LODs [126]. LODs from 0.06–2.98 ng/mL blood were described for 16 tryptamines but sensitivities for some metabolites were not included in the method of Adamowicz et al. [117]. Michely et al. [102] achieved the same LOD for 5-MeO-DALT as Ambach et al. [114], with 100 µL urine without an extraction technique, while the latter group required 250 µL urine but had a more comprehensive LLE sample cleanup. Ambach et al. also developed an analytical method for tryptamines in 10 µL dried blood spots (DBS), reaching LODs of 1–2.5 ng/mL for 5-MeO-DALT, 5-MeO-DMT, AMT, DiPT, DMT and DPT [113]. In the Brandt et al. method for 15 tryptamines, the LODs were not provided, despite their being used to propose a metabolic pathway for tryptamines [118]. Generally, the most commonly detected compounds were 5-MeO-DiPT, 5-MeO-DALT and DMT, perhaps because their metabolic pathways are already well known and toxicologists know how to identify the drugs.

## 3. Discussion

The effects of tryptamine administration are determined by their structures, as each compound has a different spectrum of receptor affinities and related psychoactive phenomena. In addition, the dose of tryptamine derivatives and duration of their effects commonly differ among compounds and depend on their potency and route of administration [127]. Given the complexity, variety and variability of the effects of psychedelics in humans, it is difficult to define animal behavior models to study psychedelic activity.

Currently, there are few data on metabolic pathways or specific enzymes involved in tryptamines biotransformation. Data suggest that not all tryptamines have a common metabolic pathway, that pathways vary based upon the nature and position of substituents in the molecule. Here we include the most current available information on tryptamines and on the reported tryptamine fatalities and intoxications. More than twenty new tryptamines were introduced to the market since the most recent reviews five years ago [22,23] and we include their data here. In addition, all the new analytical methods for tryptamines and reports of their detection in biological matrices are described. These data are valuable for physicians, toxicologists, pathologists, ED staff and others.

Despite tryptamines’ increasing consumption [1,15] recent or current use of psychedelics in general is quite low in most countries and there is limited scientific literature on their epidemiology and patterns of intake. From 2010–2020, there were few intoxication cases and even fewer fatalities due to tryptamines consumption in North America and the EU. It is unknown if there are few cases or if their detection is missed. Deaths caused by tryptamines’ consumption certainly could be missed but if they are abused alone, it is more likely that health authorities will continue the search to identify the toxicant. It is important to mention that in most cases the cause of death is not due to the toxicity of the drug but to its psychedelic effects that can lead to harmful and potentially fatal behaviors. From the fifteen case reports presented, only four resulted in a fatality [53,60,69,100]. Furthermore, three case reports [58,80,107] had no analytical confirmation, making it uncertain what drug was taken and whether the observed effects were due to the suspected drug. Tryptamines are frequently taken in combination with other drugs of abuse, for instance DMT and cannabis [33]; DMT and amphetamines [34]; 5-MeO-DiPT, cocaine and phencyclidine or codeine [100] and 5-MeO-MiPT and methylone [107], with effects resulting from the individual drugs and the interactions between drugs, based on their mechanisms of action.

The lack of knowledge of the constantly changing tryptamine availability on the internet may be driven by the lack of tryptamine testing availability. Currently, no common immunoassay-based screening test exists for tryptamine derivatives and tryptamines are not usually included in routine toxicological analyses. Therefore, wide-ranging laboratory analysis are needed for the identification of these drugs, employing techniques that can reach the required high sensitivity to avoid false negative toxicological findings and selectivity to avoid false positive tryptamine results.

Antemortem and postmortem tryptamines toxicological data most likely underestimates their prevalence because most laboratories do not routinely test for this class of drugs. Unfortunately, this results in a lack of knowledge of the drugs’ pharmacology and toxicology, clinical effects and drug-drug interactions following co-ingestions [1]. There also are few data on tryptamine and metabolite concentrations to guide toxicologists’ interpretation of tryptamine biological sample results. Currently, the use of psychedelics and anesthetic or dissociative substances remains quite low compared to other psychoactive substances but it is increasing [15].

## 4. Materials and Methods

A literature search was performed on PubMed from January 2010 to March 2020. Keywords included the names of the individual tryptamine compounds identified in laboratory casework and the published literature, general terms such as novel psychoactive substances, analytical methods, toxicology, chromatography, quantification, gas chromatography (GC), liquid chromatography (LC), psychoactive effects, pharmacology, pharmacodynamics, pharmacokinetics and drug classes including tryptamines, natural origin, synthetic origin, which were cross referenced with outcome-based terms such as overdose, intoxication, death, hospitalization, fatalities and case reports. The selection of relevant articles included the following criteria: articles acknowledging tryptamines or toxicity including studies focusing on the pharmacodynamics, analytical methods in conventional (i.e., blood, serum/plasma and urine) and alternative matrices (i.e., oral fluid and hair) and case reports published in English. Further research manuscripts were retrieved through the reference lists of selected articles and from reports and websites of international drug agencies, including the European Monitoring Centre for Drugs and Drug Addiction (EMCDDA) [128], the UNODC [129] and the World Health Organization (WHO) [130].

Three of the co-authors screened all articles independently to determine their relevance to the present review and were included if at least two co-authors selected the article. The following topics (based on title and abstract review) served to categorize each article: tryptamines pharmacology (pharmacodynamics and pharmacokinetics), chemistry, detection and identification, fatalities and intoxications. Sixty-four original research papers, thirty-one review articles and fifteen case reports were included in the review.

## Figures and Tables

**Table 1:** Chemical structures of tryptamines.

Abbreviation	Chemical Name	Molecular Formula	Chemical Structure	Molecular Weight (g/mol)	R1	R2	R3	R4	R5	R6	R7	R8
Tryptamine	Tryptamine	C_10_H_12_N_2_	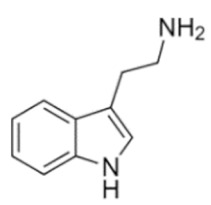	160.2	H	H	H	H	H	H	H	H
**Ring unsubstituted tryptamines**												
α-MT	α-methyltryptamine	C_11_H_14_N_2_	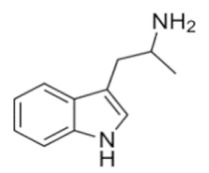	174.2	H	H	CH_3_	H	H	H	H	H
α-ET	α-ethyltryptamine	C_12_H_16_N_2_	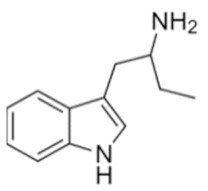	188.3	H	H	CH_2_CH_3_	H	H	H	H	H
DMT	N,N-dimethyltryptamine	C_12_H_16_N_2_	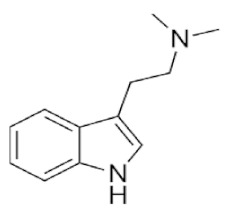	188.3	CH_3_	CH_3_	H	H	H	H	H	H
DET	N,N-diethyltryptamine	C_14_H_20_N_2_	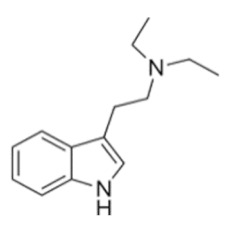	216.3	CH_2_CH_3_	CH_2_CH_3_	H	H	H	H	H	H
MET	N-methyl-N-ethyltryptamine	C_13_H_18_N_2_	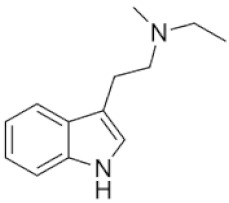	202.3	CH_3_	CH_2_CH_3_	H	H	H	H	H	H
NMT	N-methyltryptamine	C_11_H_14_N_2_	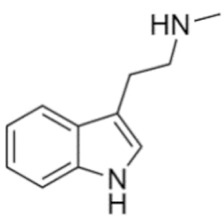	174.2	H	CH_3_	H	H	H	H	H	H
DPT	N,N-dipropyltryptamine	C_16_H_24_N_2_	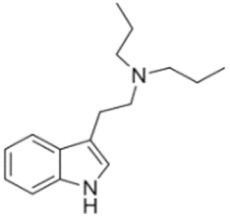	244.4	CH_2_CH_2_CH_3_	CH_2_CH_2_CH_3_	H	H	H	H	H	H
DiPT	N,N-diisopropyltryptamine	C_16_H_24_N_2_	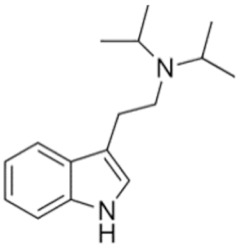	244.4	CH(CH_3_)_2_	CH(CH_3_)_2_	H	H	H	H	H	H
**4-Substituted tryptamines**												
Psilocin	4-Hydroxy-N,N-dimethyltryptamine	C_12_H_16_N_2_O	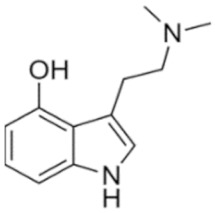	204.3	CH_3_	CH_3_	H	OH	H	H	H	H
Psilocybin	4-Phosphoryloxy-N,N-dimethyltryptamine	C_12_H_17_N_2_O_4_P	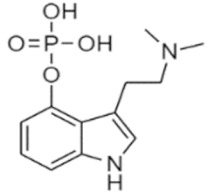	284.3	CH_3_	CH_3_	H	OPO_3_H_2_	H	H	H	H
4-OH-MET	4-Hydroxy-N-methyl-N-ethyltryptamine	C_13_H_18_N_2_O	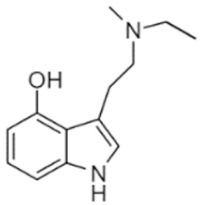	218.3	CH_3_	CH_2_CH_3_	H	OH	H	H	H	H
4-OH-DPT	4-Hydroxy-N,N-dipropyltryptamine	C_16_H_24_N_2_O	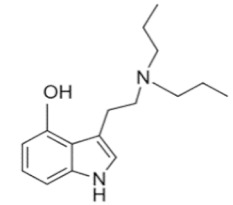	260.4	CH_2_CH_2_CH_3_	CH_2_CH_2_CH_3_	H	OH	H	H	H	H
4-OH-DET	4-Hydroxy-N,N-diethyltryptamine	C_14_H_20_N_2_O	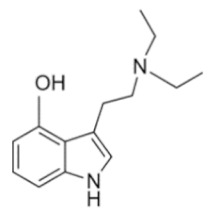	232.3	CH_2_CH_3_	CH_2_CH_3_	H	OH	H	H	H	H
4-OH-MiPT	4-Hydroxy-N-methyl-N-isopropyltryptamine	C_14_H_20_N_2_O	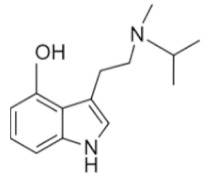	232.3	CH_3_	CH(CH_3_)_2_	H	OH	H	H	H	H
4-OH-DiPT	4-Hydroxy-N,N-diisopropyltryptamine	C_16_H_24_N_2_O	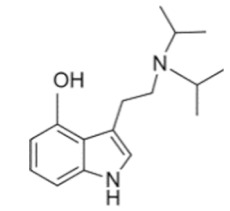	260.4	CH(CH_3_)_2_	CH(CH_3_)_2_	H	OH	H	H	H	H
4-OH-DALT	4-Hydroxy-N,N-diallyltryptamine	C_16_H_20_N_2_O	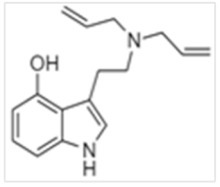	256.3	CH_2_CHCH_2_	CH_2_CHCH_2_	H	OH	H	H	H	H
4-AcO-MET	4-Acetoxy-N-methyl-N-ethyltryptamine	C_15_H_20_N_2_O_2_	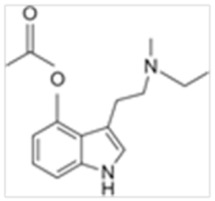	260.3	CH_3_	CH_2_CH_3_	H	OCOCH_3_	H	H	H	H
4-AcO-DPT	4-Acetoxy-N,N-dipropyltryptamine	C_18_H_26_N_2_O_2_	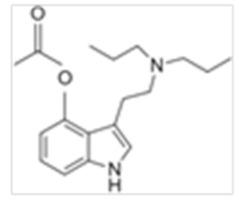	302.4	CH_2_CH_2_CH_3_	CH_2_CH_2_CH_3_	H	OCOCH_3_	H	H	H	H
4-AcO-DALT	4-Acetoxy-N,N-diallyltryptamine	C_18_H_22_N_2_O_2_	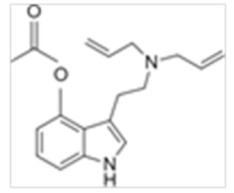	298.4	CH_2_CHCH_2_	CH_2_CHCH_2_	H	OCOCH_3_	H	H	H	H
**5-Substituted tryptamines**												
Bufotenine	5-Hydroxy-N,N-dimethyltryptamine	C_12_H_16_N_2_O	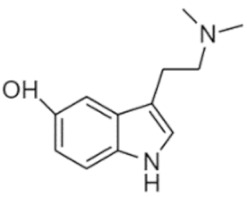	204.3	CH_3_	CH_3_	H	H	OH	H	H	H
5-OH-DiPT	5-Hydroxy-N,N-diisopropyltryptamine	C_16_H_24_N_2_O	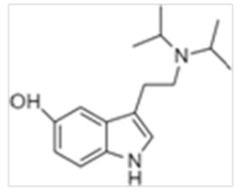	260.4	CH(CH_3_)_2_	CH(CH_3_)_2_	H	H	OH	H	H	H
5-MeO-α-MT	5-Methoxy-alpha-methyltryptamine	C_12_H_16_N_2_O	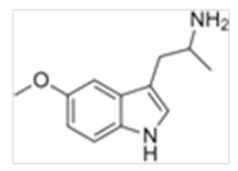	204.3	H	H	CH_3_	H	OCH_3_	H	H	H
5-MeO-DMT	5-Methoxy-N,N-dimethyltryptamine	C_13_H_18_N_2_O	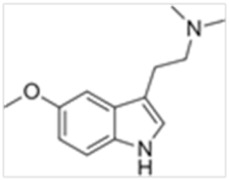	218.3	CH_3_	CH_3_	H	H	OCH_3_	H	H	H
5-MeO-DPT	5-Methoxy-N,N-dipropyltryptamine	C_17_H_26_N_2_O	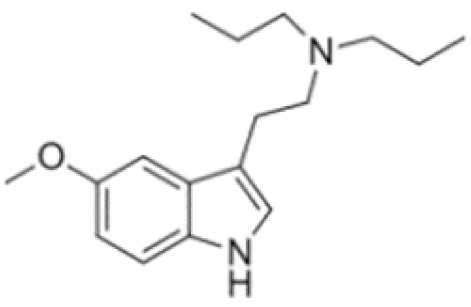	274.4	CH_2_CH_2_CH_3_	CH_2_CH_2_CH_3_	H	H	OCH_3_	H	H	H
5-MeO-DiPT	5-Methoxy-N,N-diisopropyltryptamine	C_17_H_26_N_2_O	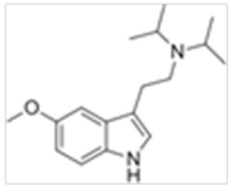	274.4	CH(CH_3_)_2_	CH(CH_3_)_2_	H	H	OCH_3_	H	H	H
5-MeO-MiPT	5-Methoxy-N-methyl-N-isopropyltryptamine	C_15_H_22_N_2_O	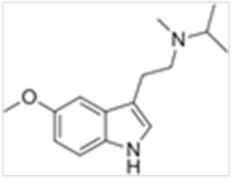	246.4	CH_3_	CH(CH_3_)_2_	H	H	OCH_3_	H	H	H
5-MeO-IPT	5-Methoxy-N-isopropyltryptamine	C_14_H_20_N_2_O	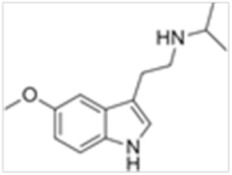	232.3	H	CH(CH_3_)_2_	H	H	OCH_3_	H	H	H
5-MeO-MET	5-Methoxy-N-methyl-N-ethyltryptamine	C_14_H_20_N_2_O	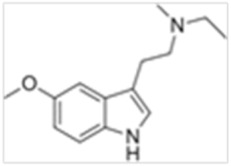	232.3	CH_3_	CH_2_CH_3_	H	H	OCH_3_	H	H	H
5-MeO-DET	5-Methoxy-N,N-diethyltryptamine	C_15_H_22_N_2_O	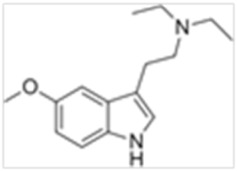	246.4	CH_2_CH_3_	CH_2_CH_3_	H	H	OCH_3_	H	H	H
5-MeO-DALT	5-Methoxy-N,N- diallyltryptamine	C_17_H_22_N_2_O	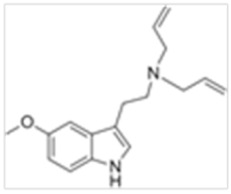	270.4	CH_2_CHCH_2_	CH_2_CHCH_2_	H	H	OCH_3_	H	H	H
5-Me-DALT	5-Methyl-N,N-diallyltryptamine	C_17_H_22_N_2_	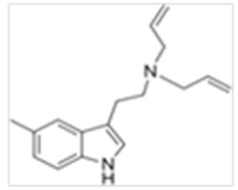	254.4	CH_2_CHCH_2_	CH_2_CHCH_2_	H	H	CH_3_	H	H	H
5-F-DALT	5-Fluoro-N,N-diallyltryptamine	C_16_H_19_N_2_F	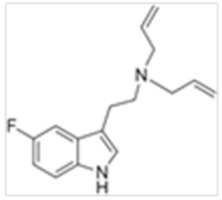	258.3	CH_2_CHCH_2_	CH_2_CHCH_2_	H	H	F	H	H	H
5-Cl-DALT	5-Chloro-N,N-diallyltryptamine	C_16_H_19_N_2_Cl	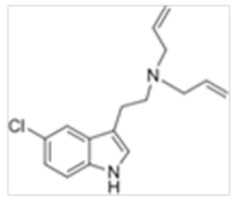	274.8	CH_2_CHCH_2_	CH_2_CHCH_2_	H	H	Cl	H	H	H
5-Br-DALT	5-Bromo-N,N-diallyltryptamine	C_16_H_19_N_2_Br	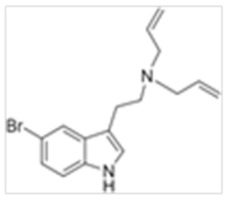	319.2	CH_2_CHCH_2_	CH_2_CHCH_2_	H	H	Br	H	H	H
5-MeO-2-Me-DALT	5-Methoxy-2-methyl-N,N-diallyltryptamine	C_18_H_24_N_2_O	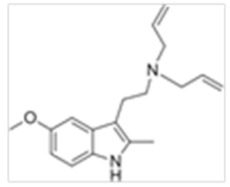	284.4	CH_2_CHCH_2_	CH_2_CHCH_2_	H	H	OCH_3_	H	H	CH_3_
5-EtO-DALT	5-Ethoxy-N,N-diallyltryptamine	C_18_H_24_N_2_O	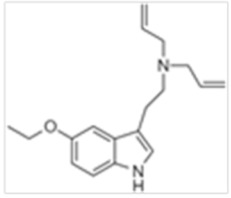	284.4	CH_2_CHCH_2_	CH_2_CHCH_2_	H	H	OCH_2_CH_3_	H	H	H
**Others**												
5,6-MD-DALT	5,6-Methylenedioxy-N,N-diallyltryptamine	C_17_H_20_N_2_O_2_	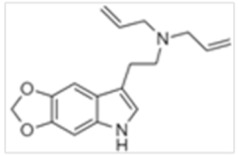	284.4	CH_2_CH	CH_2_CH	H	H	OCH_2_O *		H	H
7-Et-DALT	7-Ethyl-N,N-diallyltryptamine	C_18_H_24_N_2_	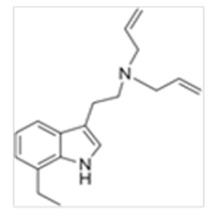	268.4	CH_2_CHCH_2_	CH_2_CHCH_2_	H	H	H	H	CH_2_CH_3_	H
7-Me-DALT	7-Methyl-N,N-diallyltryptamine	C_17_H_22_N_2_	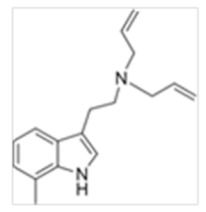	254.4	CH_2_CHCH_2_	CH_2_CHCH_2_	H	H	H	H	CH_3_	H

* Both R_5_ and R_6_ groups take part of the methylenedioxy ring.

**Table 2:** Analytical methods to determine tryptamines in conventional and non-conventional biological matrices.

Analytes	Sample (µL)	Sample Preparation	Method	Mobile Phase	Linear Range (ng/mL)	LOD (ng/mL)	LOQ (ng/mL)	Recovery (%)	Accuracy (%)	Intraday Precision (%)	Interday Precision (%)	Concentration (ng/mL)	References
5-MeO-DMT α-MT DiPT DMT DPT MiPT	P: 1000	Dilute; SPE; Evaporate; Resuspend	LC-MS/MS (positive ESI; MRM)	A: 1mM AmFo with 0.1% FA; B: MeOH with 0.1% FA	NA	1.0 2.5 1.0 2.5 1.0 2.5	NA	NA	NA	NA	NA	NA	Wohlfarth, 2010 [109]
DMT	B: 1000	Dilute; SPE; Evaporate; Resuspend	LC-QTrap/MS (positive ESI; MRM)	A: 5 mM AmFo with 0.1% FA; B:MeOH with 0.1% FA	NA	0.1	0.2	94.0–102.9	83.2–95.1	3.7–9.3	2.5–14.1	1.2–19.8	Oliveira, 2012 [110]
Psilocin	P: 500	Dilute; Centrifuge; SPE; Evaporate; Resuspend	LC-MS/MS (positive EI; MRM)	A: MeOH with 0.1% FA; B: 2 mM AmAc with 0.1% FA	2–100	0.1	0.3	86	−8.8–4.1	1.5–2.7	2.4–4.3	NA	Martin, 2012 [111]
Psilocin Bufotenine	S: 1000	Dilute; Centrifuge	LC-MS/MS (positive EI; MRM)	A: ACN with 0.1% FA; B: 2 mM AmAc buffer with 0.1% FA (pH3)	1–17.5	0.05 0.05	0.17 0.1	95 91.6	−2.9–−1.1 −3.1–−0.6	1.16–1.68 3–4.3	2.27–4.5 5.43–6.52	0.2–0.5 0.5	Martin, 2013 [112]
Psilocin Bufotenine	P: 1000	Dilute; Centrifuge	LC-MS/MS (positive EI; MRM)	A: ACN with 0.1% FA; B: 2 mM AmAc buffer with 0.1% FA (pH3)	1–17.5	0.05 0.07	0.15 0.27	93.5 91.3	−2.9–−1.1 −3.1–−0.6	1.16–1.68 3–4.3	2.27–4.5 5.43–6.52	NA	Martin, 2013 [112]
Psilocin Bufotenine	U: 500	Enzymatic hydrolysis; Centrifuge; SPE;Evaporate; Resuspend	LC-MS/MS (positive EI; MRM)	A: ACN with 0.1% FA; B: 2 mM AmAc buffer with 0.1% FA (pH3)	1–17.5	0.2 0.1	0.2 0.14	91.8 88.8	−2.9–−1.1 −3.1–−0.6	1.16–1.68 3–4.3	2.27–4.5 5.43–6.52	11–13 1.6–5.9	Martin, 2013 [112]
5-MeO-DALT 5-MeO-DMT α-MT DiPT DMT DPT	B: 10	Dilute; DBS; Evaporate; Resuspend.	LC-MS/MS (positive ESI; MRM)	A: water with 0.1% FA; B: ACN with 0.1% FA;	NA	1.0 1.0 2.5 1.0 1.0 1.0	NA	NA	NA	NA	NA	NA	Ambach, 2014 [113]
5-MeO-DALT 5-MeO-DMT α-MT DiPT DMT DPT	B: 500	Dilute; Centrifuge; SPE; Wash; Evaporate; Resuspend.	LC-MS/MS (positive ESI; sMRM)	A: 10 mM AmFo with 0.1% FA; B: MeOH with 0.1% FA	2.5–1000	NA	2.5 2.5 2.5 2.5 52.5	NA	NA	NA	NA	NA	Ambach, 2015 [114]
5-MeO-DALT 5-MeO-DMT α-MT DiPT DMT DPT	U: 250	LLE: Dilute; Centrifuge; Organic phase: evaporated; Residue: resuspended.	LC-MS/MS (positive ESI; sMRM)	A: 10 mM AmFo with 0.1% FA; B: MeOH with 0.1% FA			1 1 2.5 1 2.5 1	NA	NA	NA	NA	NA	Ambach, 2015 [114]
5-MeO-DALT	B: 500	Protein precipitation; DLLME; Centrifuge; Resuspend	LC–MS/MS (positive ESI; sMRM)	A:5mM AmFo with 0.1% AF; B: MeOH with 0.1% FA	2–1000	0.2–2	NA	5–110	NA	NA	NA	NA	Odoardi, 2015 [115]
Psilocin Bufotenine	H: 50 mg	Wash; Centrifuge; Resuspend	LC-MS/MS (positive EI; MRM)	A: ACN with 0.1% FA; B: 2 mM AmAc buffer with 0.1% FA	0.04–2	10 pg/mg	16 pg/mg 22 pg/mg	NA	−2.5–−0. 54–8.8	0.8–2.47 4.8–8.3	1.78–2.91 6.7–9.6	0.14–1.45	Martin, 2015 [116]
DALT 5-MeO-DALT	U:100	Dilute; Centrifuge; Evaporate; Resuspend	LC-HR-MS/MS (positive ionization; full scan & DDA)	A: 2 mM AmFo with 0.1% FA; B: 2 mM AmFo : ACN:MeOH (50:50, *v/v*; 1% water) with 0.1% FA	NA	1	NA	NA	NA	NA	NA	NA	Michely, 2015 [102]
DALT 5-MeO-DALT	U: 5000	Acid hydrolysis	GC–MS SUSA (positive EI; full scan)	helium	NA	10 50	NA	NA	NA	NA	NA	NA	Michely, 2015 [102]
4-OH-DET 4-OH-DIPT 4-OH-MET 4-OH-MIPT 4-MeO-DMT 4-AcO-DiPT 5-MeO-α-MT 5-Meo-DALT 5-MeO-DiPT 5-MeO-DMT 5-MeO-MiPT α-MT DiPT DMT DPT MET NMT DET	B: 200	Dilute; Protein precipitation; Centrifuge; Evaporate; Resuspend	LC-MS/MS (positive ESI; dMRM)	A: ACN with 0.1% FA; B: water with 0.1% FA	1–100	0.06 1.05 NA NA 2.98 0.99 0.11 1.11 0.15 2.61 NA 2.61 0.88 0.08 0.30 NA 1.50 0.72	NA	17.1	DMT:111	DMT:7	DMT: 10.3	DMT:11.1	Adamowicz, 2016 [117]
DALT 2-Ph-DALT 4-AcO-DALT 4-OH-DALT 5-Me-DALT 5-MeO-DALT 5-MeO-2-Me-DALT 5-EtO-DALT 5-F-DALT 5-F-2-Me-DALT 5-Cl-DALT 5-Br-DALT 6-F-DALT 7-Me-DALT 7-Et-DALT	B, P, U: NA	Dilute and shoot	GC-MS; sIR; (positive ESI; full scan & PDA)	helium	NA	NA	NA	NA	NA	NA	NA	NA	Brandt, 2016 [118]
DALT 2-Ph-DALT 4-AcO-DALT 4-OH-DALT 5-Me-DALT 5-MeO-DALT 5-MeO-2-Me-DALT 5-EtO-DALT 5-F-DALT 5-F-2-Me-DALT 5-Cl-DALT 5-Br-DALT 6-F-DALT 7-Me-DALT 7-Et-DALT	B, P, U: NA	Dilute and shoot	LC-MS; sIR; (positive HESI; full scan & DDA)	A: water with 0.1% FA; B: ACN with 0.1% FA	NA	NA	NA	NA	NA	NA	NA	NA	Brandt, 2016 [118]
5-MeO-DiPT 4-OH-DiPT	B: 200	Protein precipitation; Centrifuge; Evaporate; Resuspend	LC-MS/MS (positive ESI; MRM; full scan & DDA)	A: 5 mM FA B: ACN	1–100	0.1	0.3	84 91	−13.3–6.3 7.7–14.7	−9.9–14.9 2.2–15.7	9.7–14.6 6.9–14.1	NA	Vaiano, 2016 [119]
DMT 5-MeO-DMT α-MT	OF: 500	Dilution; LLE; Centrifuge; Evaporate; Resuspend	UHPLC-MS/MS (positive ESI; MRM)	A:10mM AmFo B: meOH	NA	NA	NA	NA	NA	NA	NA	NA	Gjerde, 2016 [120]
5-MeO-MiPT	S: 100	Dilute; Centrifuge; Evaporate; Resuspend	UHPLC-HRMS XEVO G2 QTOFMS (positive ESI)	A: water with 0.01% FA; B:MeOH with 0.01% FA	NA	NA	NA	NA	NA	NA	NA	NA	Fabregat-Safont, 2017 [121]
5-MeO-MiPT	U: 200	Enzymatic hydrolysis; Protein precipitation; Centrifuge	UHPLC-HRMS interfaced to a XEVO G2 QTOF MS(positive ESI)	A: water with 0.01% FA; B:MeOH with 0.01% FA	NA	NA	NA	NA	NA	NA	NA	NA	Fabregat-Safont, 2017 [121]
5-MeO-MiPT	U: 100	Protein precipitation; Centrifuge	UHPLC-HRMS interfaced to a XEVO G2 QTOF MS (positive ESI)	A: water with 0.01% FA; B:MeOH with 0.01% FA	NA	NA	NA	NA	NA	NA	NA	NA	Fabregat-Safont, 2017 [121]
5-F-DALT 7-Me-DALT 6-MD-DALT	U: 100	Dilute; Centrifuge; Evaporate; Resuspend	LC-HRMS/MS (positive HESI; full scan & DDA	A: 2 mM aqueous AmFo with 0.1% FA (pH 3); B: 2 mM aqueous AmFo with ACN:MeOH (1:1, v/v; 1% water) with 0.1% FA	NA	NA	NA	NA	NA	NA	NA	NA	Michely, 2017 [122]
5-F-DALT 7-Me-DALT 5,6-MD-DALT	U: 2500	Acid hydrolysis	GC-MS SUSA (positive ESI; full scan)	helium	NA	NA	NA	NA	NA	NA	NA	NA	Michely, 2017 [122]
Psilocin DMT 5-MeO-DMT	B: 1000	Centrifuge; Evaporate; Resuspend	LC-MS/MS (positive ESI; dMRM)	A: 5mM AmFo in water with 0.01% FA; B: ACN with 0.01% FA	NA	2.50	NA	NA	NA	NA	NA	NA	Fagiola, 2018 [123]
Psilocin DMT 5-MeO-DMT	U: 1000	Centrifuge; Evaporate; Resuspend	LC-MS/MS (positive ESI; dMRM)	A: 5mM AmFo in water with 0.01% FA; B: ACN with 0.01% FA	NA	2.50	NA	NA	NA	NA	NA	NA	Fagiola, 2018 [123]
5-MeO-DiPT	H: 30 mg	Wash; Pulverize; Centrifuge	LC-MS/MS (positive ESI; MRM)	A: 20 mM AmAc, 5% ACN and water with 0.1% FA; B: ACN with 0.1% FA	0.1–100 pg/mg	NA	0.1 pg/mg	91.1–112	92.1–106	<13	12.1–17.0	0.2–7533 pg/mg	Roujia, 2019 [124]
DMT Psilocybin	U: NA	Dilute	LC-QTOF (positive ESI)	A: 5 mM AmFo (pH 3) B: ACN with 0.1% AF	NA	1–2	NA	NA	NA	NA	NA	NA	Pope, 2019 [125]
5-MeO-DiPT 5-OH-DiPT 5-MeO-IPT	U:1000	Centrifuge; Resuspend	GC- Orbitrap- MS (positive EI; full scan)	helium	2–300	1	2	92.4–98.4	93–108.7	3.1–7.2	7.2–10.3	2–2.8	Yan, 2020 [126]

**Abbreviations:** 5-MeO-DMT, 5-Methoxy-N,N-dimethyltryptamine; α-MT, α-methyltryptamine; DiPT, N,N-diisopropyltryptamine; DMT, N,N-dimethyltryptamine; DPT, N,N-dipropyltryptamine; DET; N,N-diethyltryptamine; MiPT, N-methyl-N-isopropyltryptamine; 5-MeO-DALT, 5-Methoxy-N,N-diallyltryptamine; DPT, N,N-dipropyltryptamine; 4-OH-DET, 4-Hydroxy-N,N-diethyltryptamine; 4-OH-DIPT, 4-Hydroxy-N,N-diisopropyltryptamine; 4-AcO-DiPT, 4-Acetoxy- N,N-diisopropyltryptamine; 4-OH-MET, 4-Hydroxy-N-methyl-N-ethyltryptamine; 4-OH-MIPT, 4-Hydroxy-N-methyl-N-isopropyltryptamine; 4-MeO-DMT, 4-Methoxy-N,N-dimethyltryptamine; 5-MeO-α-MT, 5-Methoxy-alfa-methyltryptamine; 5-MeO-DiPT, 5-Methoxy-N,N-diisopropyltryptamine; 5-MeO-DMT, 5-Methoxy-N,N-dimethyltryptamine; 5-MeO-MiPT, 5-Methoxy-N-methyl-N-isopropyltryptamine; MET, N-methyl-N-ethyltryptamine; DET, N,N-diethyltryptamine; NMT, N-methyltryptamine; DALT, N,N-diallyltryptamine; 5-MeO-DALT, 5-Methoxy-N,N-diallyltryptamine; 2-Ph-DALT, 2-Phenyl-N,N-diallyltryptamine; 4-AcO-DALT, 4-Acetoxy-N,N-diallyltryptamine; 4-OH-DALT, 4-Hydroxy-N,N-diallyltryptamine; 5-Me-DALT, 5-Methyl-N,N-diallyltryptamine; 5-MeO-2-Me-DALT, 5-Methoxy-2-methyl-N,N-diallyltryptamine; 5-EtO-DALT, 5-Ethoxy-N,N-diallyltryptamine; 5-F-DALT, 5-Fluoro-N,N-diallyltryptamine; 5-F-2-Me-DALT, 5-Fluoro-2-methyl-N,N-diallyltryptamine; 5-Cl-DALT, 5-Chloro-N,N-diallyltryptamine; 5-Br-DALT, 5-Bromo-N,N-diallyltryptamine; 6-F-DALT, 6-Fluoro-N,N-diallyltryptamine; 7-Me-DALT, 7-Methyl-N,N-diallyltryptamine; 7-Et-DALT, 7-Ethyl-N,N-diallyltryptamine; 6-MD-DALT, 6-Methylenedioxy-N,N-diallyltryptamine; 5,6-MD-DALT, 5, 6-Methylenedioxy-N,N-diallyltryptamine; 5-MeO-IPT, 5-Methoxy-isopropyltryptamine; H, hair; B, blood; U, urine; S, serum; P, plasma; SPE, solid phase extraction; LLE, liquid liquid extraction; DLLME, dispersive liquid-liquid microextraction; DBS, dried blood spots; UPLC, ultra-performance liquid chromatography; LC-MS/MS, liquid chromatography coupled with mass spectrometry in tandem; LC- HRMS/MS, liquid chromatography high resolution coupled with mass spectrometry in tandem; DDA, data dependent acquisition; PDA, photodiode array acquisition; sMRM, scheduled multiple reaction monitoring; dMRM, dynamic multiple reaction monitoring; QTrap, triple quadrupole linear ion trap mass spectrometer; UHPLC-HRMS, ultra-performance liquid chromatography-high resolution mass spectrometry; GC-Orbitrap-MS, gas chromatography coupled with Orbitrap mass spectrometry; HPLC, high performance liquid chromatography; UHPLC, ultra-high performance liquid chromatography; LC-QTOF, liquid chromatography coupled to a Quadrupole Time of Flight mass spectrometer; GC-MS, gas chromatography coupled with mass spectrometry; ESI, electrospray ionization; HESI, heated electro spray ionization; EI, electron ionization; MRM, multiple reaction monitoring; GC-sIR, Gas chromatography solid-state infrared analysis; AmAc, ammonium acetate; FA, formic acid; ACN, acetonitrile; AmFo, ammonium formate; MeOH, methanol; LOD, lower limit of detection; LOQ, limit of quantification; NA, not available.

## Abbreviations

2-MTHβC	2-Methyl-1,2,3,4-tetrahydro-beta-carboline
4-AcO-DALT	4-Acetoxy-N, N-diallyltryptamine
4-AcO-DET	4-Acetoxy-N,N-diethyltryptamine
4-AcO-DiPT	4-Acetoxy-N, N- diisopropyltryptamine
4-AcO-DMT	4-Acetoxy-N,N-dimethyltryptamine
4-AcO-DPT	4-Acetoxy-N, N-dipropyltryptamine
4-AcO-MET	4-Acetoxy-N-methyl-N-ethyltryptamine
4-HIAA	4-Hydroxyindole acetic acid
4-MeO-DMT	4-Methoxy-N,N-dimethyltryptamine
4-MeO-MiPT	4-Methoxy-N-methyl-N-isopropyltryptamine
4-OH-DET	4-Hydroxy-diethyl-tryptamine
4-OH-DMT	4-Hydroxy-NN-dimethyltryptamine
4-OH-DPT	4-Hydroxy-N,N-dipropyltryptamine
4-OH-MET	4-Hydroxy-N-methyl-N-ethyltriptamine
4-OH-MiPT	4-Hydroxy-N-methyl-N-isopropyltryptamine
4-OH-MPT	4-Hydroxy-N-methyl-N-propyltryptamine
4-OH-pyr-T	4-Hydroxy-N,N-tetramethylenetryptamine
5-HT	5-Hydroxytryptamine
5-MeO-DALT	5-Methoxy-N,N-diallyltryptamine
5-MeO-DET	5-Methoxy-N,N-diethyltryptamine
5-MeO-DiPT	5-Methoxy-diisopropyltryptamine
5-MeO-DMT	5-Methoxy-N,N-dimethyltryptamine
5-MeO-DPT	5-Methoxy-N, N-dipropyltryptamine
5-MeO-MET	5-Methoxy-N-methyl-N-ethyltryptamine
5-MeO-MiPT	5-Methoxy-N-methyl-N-isopropyltryptamine
5-MeO-MiPT-N-oxide	5-Methoxy-N-methyl-N-isopropyltrypt-amine-N-oxide
5-MeO-NiPT	5-Methoxy-N-isopropyltryptamine
5-MeO-TMT	5-Methoxy-N,N-trimethyltryptamine
5-MeO-α-MT	5-Methoxy-alpha-methyltryptamine
5-OH-DiPT	5-Hydroxy-N,N-diisopropyltryptamine
5-OH-DMT	5-Hydroxy-N,N-dimethyltryptamine
5-OH-MiPT	5-Hydroxy-N-methyl-N-isopropyltryptamine
CNS	Central nervous system
DBS	Dried blood spots
DDA	Data dependent acquisition
DEA	Drug Enforcement Administration
DET	N,N-diethyltryptamine
DiPT	N,N-diisopropyltryptamine
DLLME	Dispersive liquid-liquid microextraction
DMT	N,N-dimethyltryptamine
DMT-NO	Dimethyltryptamine-N-oxide
DPT	N,N-dipropyltryptamine
ED	Emergency department
EMCDDA	European Monitoring Centre for Drugs and Drug Addiction
FDA	Food and Drug Administration
GC	Gas chromatography
GC-EI-TOF	Gas chromatography time-of-flight detection with electron impact ionization
GC-MS	Gas chromatography-mass spectrometry
GDS	Global Drug Survey
HPLC	High-performance liquid chromatography
HPLC-MS	High-performance liquid chromatography coupled with mass spectrometry
IAA	Indole-3-Acetic Acid
IM	Intramuscular
IV	Intravenous
LC	Liquid chromatography
LC–HRMS/MS	Liquid chromatography–high-resolution mass spectrometry
LC-MS/MS	Liquid chromatography coupled with mass spectrometry in
LLE	Liquid-liquid extraction
LOD	Limit of detection
LSD	Lysergic acid diethylamide
MAO	Monoamine oxidase
MAOI	Monoamine oxidase inhibitor
MDMA	3,4-Methylenedioxymethamphetamine
MET	N-methyl-N-ethyltryptamine
MRM	Multiple reaction monitoring
NMT	N-methyltryptamine
NPS	New psychoactive substances
OH-5-MeO- MiPT	hydroxy-5-methoxy-N-methyl-N-isopropyl-tryptamine
PDA	Photodiode array acquisition
SIM	Single ion monitoring
SPE	Solid phase extraction
STAI	Speilberg State-Trait Anxiety Inventory
TOF	Time of flight
UHPLC-HRMS/MS	Ultra-performance liquid chromatography-high resolution mass spectrometry
UNODC	United Nations Office on Drugs and Crime
WHO	World Health Organization
α-ET	Alpha-ethyltryptamine
α-MT	Alpha-methyltryptamine
